# 2-[3-(Methyl­diphenyl­silyl)prop­yl]isoindoline-1,3-dione

**DOI:** 10.1107/S1600536809054117

**Published:** 2009-12-24

**Authors:** Ilia A. Guzei, Lara C. Spencer, Uzma I. Zakai, Daniel C. Lynch

**Affiliations:** a Department of Chemistry, University of Wisconsin–Madison, 1101 University Ave, Madison, Wisconsin 53706, USA

## Abstract

In the title compound, C_24_H_23_NO_2_Si, the dihedral angle between the planes of the phenyl rings attached to the Si atom is 80.78 (10)°. In the crystal, the mol­ecules form sheets lying perpendicular to [101] *via* C—H⋯O inter­actions. These sheets are stacked and linked in a three-dimensional framework by additional C—H⋯O inter­actions in the [10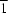] direction.

## Related literature

For literature related to drug design see: Bains & Tacke (2003 ▶); Gately & West (2007 ▶); Guzei *et al.* (2010*a* ▶,*b* ▶); Lee *et al.* (1996 ▶); Tsuge *et al.* (1985 ▶); Yoon *et al.* (1991 ▶); Zakai *et al.* (2010 ▶). For a description of the Cambridge Structural Database, see: Allen (2002 ▶). Bond distances and angles were confirmed to be typical by a *Mogul* structural check (Bruno *et al.*, 2002 ▶).
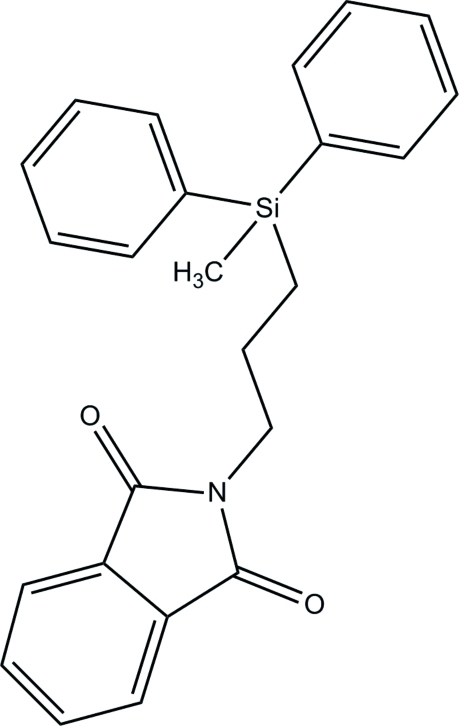


## Experimental

### 

#### Crystal data

C_24_H_23_NO_2_Si
*M* *_r_* = 385.52
Monoclinic, 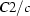

*a* = 19.277 (3) Å
*b* = 13.238 (2) Å
*c* = 19.272 (3) Å
β = 116.987 (6)°
*V* = 4382.5 (12) Å^3^
*Z* = 8
Mo *K*α radiation
μ = 0.13 mm^−1^
*T* = 300 K
0.30 × 0.30 × 0.30 mm

#### Data collection

Bruker SMART X2S diffractometer
Absorption correction: multi-scan (*SADABS*; Bruker, 2009 ▶) *T*_min_ = 0.964, *T*_max_ = 0.964
15551 measured reflections
4470 independent reflections
2693 reflections with *I* > 2σ(*I*)
*R*_int_ = 0.051

#### Refinement

*R*[*F* ^2^ > 2σ(*F* ^2^)] = 0.053
*wR*(*F* ^2^) = 0.175
*S* = 1.02
4470 reflections
254 parameters
H-atom parameters constrained
Δρ_max_ = 0.20 e Å^−3^
Δρ_min_ = −0.22 e Å^−3^

### 

Data collection: *APEX2* and *GIS* (Bruker, 2009 ▶); cell refinement: *SAINT* (Bruker, 2009 ▶); data reduction: *SAINT*; program(s) used to solve structure: *SHELXTL* (Sheldrick, 2008 ▶); program(s) used to refine structure: *SHELXTL*, *OLEX2* (Dolomanov *et al.*, 2009 ▶) and *FCF_filter* (Guzei, 2007 ▶); molecular graphics: *SHELXTL*; software used to prepare material for publication: *SHELXTL*, *modiCIFer* (Guzei, 2007 ▶) and *publCIF* (Westrip, 2010 ▶).

## Supplementary Material

Crystal structure: contains datablocks global, I. DOI: 10.1107/S1600536809054117/zs2024sup1.cif

Structure factors: contains datablocks I. DOI: 10.1107/S1600536809054117/zs2024Isup2.hkl

Additional supplementary materials:  crystallographic information; 3D view; checkCIF report

## Figures and Tables

**Table 1:** Hydrogen-bond geometry (Å, °)

*D*—H⋯*A*	*D*—H	H⋯*A*	*D*⋯*A*	*D*—H⋯*A*
C4—H4⋯O2^i^	0.93	2.63	3.366 (4)	137
C14—H14*A*⋯O1^ii^	0.97	2.63	3.582 (3)	169
C19—H19⋯O2^iii^	0.93	2.51	3.310 (3)	144
C22—H22⋯O1^iv^	0.93	2.32	3.200 (3)	157

Symmetry codes: (i) 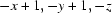; (ii) 
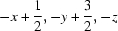
; (iii) 
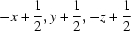
; (iv) 
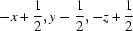
.

**Fig. 1.**
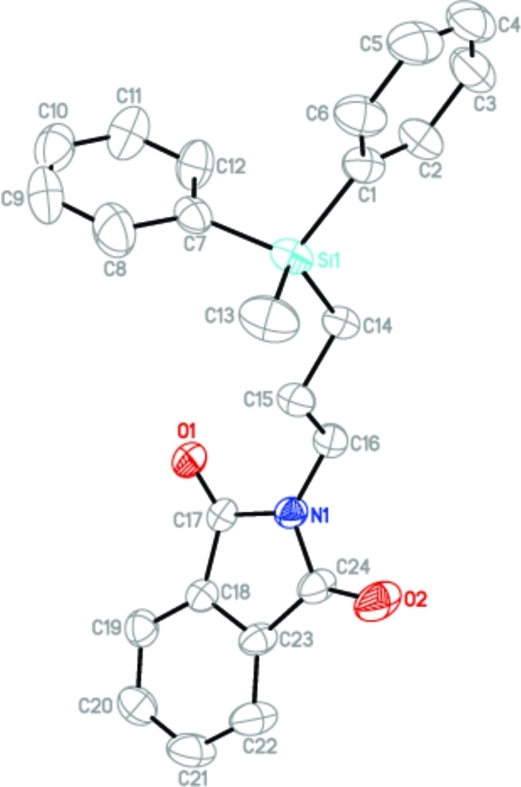
Molecular structure of (I). The thermal ellipsoids are shown at 50% probability level.

## supplementary crystallographic information

### Comment

Sila phthalimides have been used in photocyclization reactions (Yoon *et al.*, 1991), as protecting groups stabilizing reactive intermediates (Tsuge *et al.*, 1985), and as reactants that undergo intramolecular hydrogen-abstraction (Lee *et al.*, 1996). In our laboratory the title compound C_24_H_23_NO_2_Si (I) was isolated as an intermediate in the synthesis of the respective sila amine. It is a congener of 2-(((4-methoxyphenyl)dimethylsilyl)methyl)isoindoline-1,3-dione (Guzei *et al.*, 2010*a*) and 2-(2-(trimethylsilyl)ethyl)isoindoline-1,3-dione (Guzei *et al.*, 2010*b*) recently reported by us. These sila amines were subsequently coupled with a selection of pharmaceutical agents containing a carboxylic acid, including indomethacin and *N*-acetyl *L*-cysteine (Zakai *et al.*, 2010) as part of our continuing efforts at drug repurposing (Gately & West, 2007) using silicon chemistry (Bains & Tacke, 2003).

In the structure of (I) the bond distances and angles are typical as confirmed by the *Mogul* structural check (Bruno *et al.*, 2002). The average Si—C distance of 1.870 (3) Å for compound (I) is statistically similar to the 1.88 (2) Å average of 41 measurements for 10 related compounds in the Cambridge Structural Database (CSD; Version 1.11, September 2009 release; Allen, 2002). The Si atom exhibits a slightly imperfect tetrahedral geometry with angles ranging from 108.56 (10)° to 110.53 (12)°. The phthalate entity is expectedly planar within 0.0085 Å. The two phenyl groups exhibit a windmill-like geometry about the central silicon atom. The planes of the two phenyl groups form an angle of 80.78 (10)°. For 33 compounds in the CSD that have a central silicon atom with a methyl group, two phenyl groups and another arbitrary group attached, the planes between the two phenyl groups averaged 73 (9)°, similar to that of (I).

Each oxygen atom participates in two C—H···O interactions (Table 1) which help form the three-dimensional structure of (I). These weak interactions involving O2 form sheets of (I) perpendicular to [1 0 1]. These sheets are stacked and linked in the three-dimensional framework by the interactions involving O1 in the [1 0 1] direction.

### Experimental

The protocol described by Tsuge and co-workers (Tsuge *et al.*, 1985) was adopted. The required amount of potassium phthalimide (7.42 g, 40 mmol, 1.1 equiv) was placed into a 250 ml round-bottom flask, which was then sealed and flushed with nitrogen three times. Dry DMF (56 ml) was syringed into the flask followed by the addition of chloropropyldiphenylmethylsilane (10 g, 36.46 mmol, 1 equiv.) The reaction was heated at 60°C for 6 h. The resulting mixture was allowed to cool to room temperature. This slurry was poured onto a minimal quantity of water and extracted 3–5 times with diethyl ether. The organic extracts were subsequently collected, dried with magnesium sulfate, and filtered. The filtrate was mixed with silica gel and evaporated under reduced pressure to afford a powder of silica gel. This powder was then loaded onto a dry-packed silica gel column and eluted using a gradient column. The fractions of interest were usually drawn out using a 8:2 hexane:ethyl acetate mixture. The fractions were then combined to afford the title compound. Further recrystallization from dichloromethane afforded large lusterous white crystals (12.47 g, 32.35 mmol, 89% yield) for X-ray crystallography. Manipulation of air and moisture sensitive compounds was performed using standard high-vacuum line techniques. All solvents and reagents were obtained from Aldrich. Chloropropyldiphenylmethylsilane was purchased from Gelest. ^1^H NMR spectra were obtained on a Varian Unity 500 spectrometer, ^13^C {H} NMR spectra were obtained on a Varian 500 spectrometer operating at 125 MHz, ^29^Si {H} NMR spectra were obtained on a Varian Unity spectrometer operating at 99 MHz. Mass spectra were determined on a Waters (Micromass) AutoSpec mass spectrometer. Melting points were determined on a Mel-Temp Laboratory Device. mp 57–58° C; ^1^H NMR (500 MHz, CDCl_3_) δ 0.52 (s, 3H, Me), 1.08 (m, 2H, CH_2_), 1.73 (m, 2H, CH_2_), 3.67 (t, *J*=7.3 Hz, 2H, CH_2_), 7.33 (m, 6H, ArH), 7.47 (dd, *J*=7.4, 1.5 Hz, 4H, ArH), 7.68 (dd, *J*=5.5, 3.0 Hz, 2H, ArH), 7.80 (dd, *J*=5.4, 3.1 Hz, 2H, ArH); ^13^C NMR (125 MHz, CDCl_3_) δ -4.6 (SiCH_3_), 11.5 (CH_2_), 23.2 (CH_2_), 40.9 (CH_2_), 123.1 (CH), 127.9 (CH), 129.2 (CH), 132.1 (CH), 133.8 (CH), 134.4 (CH), 136.6 (CH), 168.4 (CO); ^29^Si NMR (99 MHz, CDCl_3_) δ -7.62 (SiMePh_2_); MS (EI^+^) *m/z* (*rel*. intensity %) 385 (*M*^+^, 5), 370 (M—Me, 21), 308 (100), 266 (70), 197 (96), 160 (62): HRMS (EI^+^): calcd. for C_24_H_23_NO_2_Si (*M*^+^) 385.1493, found (M—Me)^+^ 370. 1258.

### Refinement

All H-atoms were placed in idealized locations and refined as riding with appropriate thermal displacement coefficients *U*_iso_(H) = 1.2 or 1.5 times *U*_eq_(bearing atom). The data were collected at room temperature on a Bruker SMART X2S diffractometer in the automated mode and manually processed thereafter.

### Crystal data

**Table:**

C_24_H_23_NO_2_Si	*F*(000) = 1632
*M**_r_* = 385.52	*D*_x_ = 1.169 Mg m^−^^3^
Monoclinic, *C*2/*c*	Mo *K*α radiation, λ = 0.71073 Å
Hall symbol: -C 2yc	Cell parameters from 3839 reflections
*a* = 19.277 (3) Å	θ = 2.4–22.8°
*b* = 13.238 (2) Å	µ = 0.13 mm^−^^1^
*c* = 19.272 (3) Å	*T* = 300 K
β = 116.987 (6)°	Block, colourless
*V* = 4382.5 (12) Å^3^	0.30 × 0.30 × 0.30 mm
*Z* = 8	

### Data collection

**Table:**

Bruker SMART X2S diffractometer	4470 independent reflections
Radiation source: micro-focus sealed tube	2693 reflections with *I* > 2σ(*I*)
doubly curved silicon crystal	*R*_int_ = 0.051
ω scans	θ_max_ = 26.5°, θ_min_ = 1.9°
Absorption correction: multi-scan (*SADABS*; Bruker, 2009)	*h* = −23→21
*T*_min_ = 0.964, *T*_max_ = 0.964	*k* = −16→16
15551 measured reflections	*l* = −20→24

### Refinement

**Table:**

Refinement on *F*^2^	Primary atom site location: structure-invariant direct methods
Least-squares matrix: full	Secondary atom site location: difference Fourier map
*R*[*F*^2^ > 2σ(*F*^2^)] = 0.053	Hydrogen site location: inferred from neighbouring sites
*wR*(*F*^2^) = 0.175	H-atom parameters constrained
*S* = 1.01	*w* = 1/[σ^2^(*F*_o_^2^) + (0.102*P*)^2^] where *P* = (*F*_o_^2^ + 2*F*_c_^2^)/3
4470 reflections	(Δ/σ)_max_ < 0.001
254 parameters	Δρ_max_ = 0.20 e Å^−^^3^
0 restraints	Δρ_min_ = −0.22 e Å^−^^3^
0 constraints	

### Special details

**Table:**

Geometry. All e.s.d.'s (except the e.s.d. in the dihedral angle between two l.s. planes) are estimated using the full covariance matrix. The cell e.s.d.'s are taken into account individually in the estimation of e.s.d.'s in distances, angles and torsion angles; correlations between e.s.d.'s in cell parameters are only used when they are defined by crystal symmetry. An approximate (isotropic) treatment of cell e.s.d.'s is used for estimating e.s.d.'s involving l.s. planes.
Refinement. Refinement of *F*^2^ against ALL reflections. The weighted *R*-factor *wR* and goodness of fit *S* are based on *F*^2^, conventional *R*-factors *R* are based on *F*, with *F* set to zero for negative *F*^2^. The threshold expression of *F*^2^ > σ(*F*^2^) is used only for calculating *R*-factors(gt) *etc*. and is not relevant to the choice of reflections for refinement. *R*-factors based on *F*^2^ are statistically about twice as large as those based on *F*, and *R*- factors based on ALL data will be even larger.

### Fractional atomic coordinates and isotropic or equivalent isotropic displacement parameters (Å^2^)

**Table:**

	*x*	*y*	*z*	*U*_iso_*/*U*_eq_	
Si1	0.49123 (4)	0.69714 (5)	0.07188 (4)	0.0625 (2)	
O1	0.22870 (10)	0.74572 (11)	0.13602 (10)	0.0738 (5)	
O2	0.28657 (14)	0.41221 (13)	0.16823 (14)	0.1108 (8)	
N1	0.26255 (10)	0.57953 (12)	0.13731 (11)	0.0566 (5)	
C1	0.52300 (14)	0.67920 (17)	−0.00584 (15)	0.0653 (6)	
C2	0.47118 (17)	0.6588 (2)	−0.08206 (16)	0.0812 (8)	
H2	0.4186	0.6520	−0.0956	0.097*	
C3	0.4956 (2)	0.6483 (2)	−0.13887 (18)	0.0995 (10)	
H3	0.4593	0.6351	−0.1900	0.119*	
C4	0.5722 (2)	0.6569 (2)	−0.1209 (2)	0.0993 (10)	
H4	0.5881	0.6496	−0.1596	0.119*	
C5	0.6249 (2)	0.6761 (2)	−0.0473 (2)	0.0980 (10)	
H5	0.6773	0.6816	−0.0349	0.118*	
C6	0.60125 (16)	0.6874 (2)	0.01005 (19)	0.0855 (8)	
H6	0.6384	0.7010	0.0608	0.103*	
C7	0.47864 (13)	0.8354 (2)	0.08261 (14)	0.0651 (6)	
C8	0.5175 (2)	0.8887 (3)	0.15175 (19)	0.1228 (12)	
H8	0.5505	0.8541	0.1967	0.147*	
C9	0.5085 (3)	0.9920 (4)	0.1554 (3)	0.1427 (17)	
H9	0.5362	1.0256	0.2025	0.171*	
C10	0.4601 (2)	1.0448 (3)	0.0918 (3)	0.1084 (12)	
H10	0.4534	1.1139	0.09050	0.130*	
C11	0.42195 (19)	0.9959 (2)	0.0240 (2)	0.1046 (10)	
H11	0.3888	1.0313	−0.0205	0.126*	
C12	0.43174 (16)	0.8933 (2)	0.01984 (18)	0.0872 (8)	
H12	0.4050	0.8617	−0.0282	0.105*	
C13	0.56729 (17)	0.6463 (3)	0.16613 (16)	0.1021 (10)	
H13A	0.6151	0.6823	0.1808	0.153*	
H13B	0.5753	0.5758	0.1603	0.153*	
H13C	0.5506	0.6545	0.2057	0.153*	
C14	0.39646 (13)	0.63048 (18)	0.04274 (12)	0.0603 (6)	
H14A	0.3568	0.6634	−0.0030	0.072*	
H14B	0.4014	0.5616	0.0284	0.072*	
C15	0.36934 (13)	0.62798 (19)	0.10561 (13)	0.0630 (6)	
H15A	0.4053	0.5873	0.1489	0.076*	
H15B	0.3704	0.6960	0.1247	0.076*	
C16	0.28795 (13)	0.58526 (18)	0.07650 (14)	0.0635 (6)	
H16A	0.2518	0.6273	0.0344	0.076*	
H16B	0.2865	0.5181	0.0557	0.076*	
C17	0.23600 (12)	0.66153 (16)	0.16301 (13)	0.0539 (5)	
C18	0.21922 (11)	0.62472 (16)	0.22621 (13)	0.0542 (5)	
C19	0.19309 (15)	0.67446 (19)	0.27256 (16)	0.0721 (7)	
H19	0.1810	0.7429	0.2658	0.086*	
C20	0.18555 (18)	0.6191 (2)	0.32939 (17)	0.0896 (8)	
H20	0.1681	0.6508	0.3616	0.108*	
C21	0.2033 (2)	0.5187 (3)	0.33913 (19)	0.0996 (10)	
H21	0.1974	0.4831	0.3777	0.120*	
C22	0.22994 (18)	0.4688 (2)	0.29304 (19)	0.0908 (9)	
H22	0.2424	0.4005	0.3002	0.109*	
C23	0.23748 (13)	0.52346 (17)	0.23615 (15)	0.0636 (6)	
C24	0.26513 (14)	0.49367 (18)	0.17894 (16)	0.0691 (7)	

### Atomic displacement parameters (Å^2^)

**Table:**

	*U*^11^	*U*^22^	*U*^33^	*U*^12^	*U*^13^	*U*^23^
Si1	0.0530 (4)	0.0846 (5)	0.0515 (4)	0.0067 (3)	0.0251 (3)	0.0098 (3)
O1	0.0954 (13)	0.0496 (10)	0.0769 (11)	0.0033 (8)	0.0396 (10)	0.0045 (8)
O2	0.158 (2)	0.0577 (11)	0.180 (2)	0.0346 (11)	0.1325 (19)	0.0264 (12)
N1	0.0634 (11)	0.0494 (10)	0.0682 (11)	0.0020 (8)	0.0397 (10)	0.0031 (9)
C1	0.0648 (15)	0.0681 (15)	0.0721 (16)	0.0150 (11)	0.0390 (13)	0.0143 (12)
C2	0.0806 (18)	0.105 (2)	0.0716 (17)	0.0091 (15)	0.0464 (15)	0.0002 (15)
C3	0.124 (3)	0.115 (2)	0.078 (2)	0.015 (2)	0.062 (2)	−0.0006 (17)
C4	0.128 (3)	0.096 (2)	0.118 (3)	0.030 (2)	0.095 (3)	0.017 (2)
C5	0.089 (2)	0.101 (2)	0.137 (3)	0.0220 (17)	0.079 (2)	0.024 (2)
C6	0.0721 (18)	0.106 (2)	0.094 (2)	0.0130 (14)	0.0510 (16)	0.0172 (16)
C7	0.0525 (13)	0.0877 (17)	0.0616 (14)	−0.0116 (11)	0.0314 (12)	−0.0121 (12)
C8	0.155 (3)	0.119 (3)	0.073 (2)	−0.012 (2)	0.033 (2)	−0.0198 (19)
C9	0.204 (5)	0.115 (3)	0.110 (3)	−0.040 (3)	0.072 (3)	−0.056 (3)
C10	0.114 (3)	0.086 (2)	0.155 (4)	−0.017 (2)	0.087 (3)	−0.035 (2)
C11	0.082 (2)	0.079 (2)	0.132 (3)	−0.0013 (16)	0.031 (2)	−0.014 (2)
C12	0.0730 (17)	0.0759 (19)	0.088 (2)	−0.0038 (14)	0.0153 (15)	−0.0160 (15)
C13	0.0762 (19)	0.147 (3)	0.0691 (18)	0.0160 (18)	0.0204 (16)	0.0311 (18)
C14	0.0647 (14)	0.0678 (14)	0.0523 (12)	0.0030 (11)	0.0300 (11)	0.0069 (10)
C15	0.0629 (14)	0.0735 (15)	0.0577 (13)	−0.0023 (11)	0.0318 (12)	0.0032 (11)
C16	0.0670 (15)	0.0629 (14)	0.0661 (15)	−0.0037 (11)	0.0349 (13)	−0.0027 (11)
C17	0.0511 (12)	0.0451 (12)	0.0595 (13)	−0.0003 (9)	0.0198 (10)	−0.0006 (10)
C18	0.0479 (12)	0.0533 (12)	0.0636 (13)	−0.0007 (9)	0.0272 (11)	−0.0002 (10)
C19	0.0773 (17)	0.0658 (15)	0.0805 (17)	0.0008 (12)	0.0422 (15)	−0.0088 (13)
C20	0.101 (2)	0.101 (2)	0.090 (2)	0.0022 (17)	0.0634 (18)	−0.0063 (17)
C21	0.117 (3)	0.112 (3)	0.101 (2)	0.0117 (19)	0.077 (2)	0.0269 (19)
C22	0.104 (2)	0.0776 (18)	0.121 (2)	0.0234 (15)	0.078 (2)	0.0353 (17)
C23	0.0628 (14)	0.0561 (13)	0.0859 (16)	0.0079 (10)	0.0461 (13)	0.0131 (12)
C24	0.0741 (16)	0.0513 (14)	0.1020 (19)	0.0119 (11)	0.0577 (15)	0.0121 (12)

### Geometric parameters (Å, °)

**Table:**

Si1—C13	1.867 (3)	C10—H10	0.9300
Si1—C7	1.870 (3)	C11—C12	1.378 (4)
Si1—C14	1.871 (2)	C11—H11	0.9300
Si1—C1	1.873 (3)	C12—H12	0.9300
O1—C17	1.211 (2)	C13—H13A	0.9600
O2—C24	1.206 (3)	C13—H13B	0.9600
N1—C24	1.379 (3)	C13—H13C	0.9600
N1—C17	1.384 (3)	C14—C15	1.522 (3)
N1—C16	1.463 (3)	C14—H14A	0.9700
C1—C2	1.377 (4)	C14—H14B	0.9700
C1—C6	1.401 (4)	C15—C16	1.516 (3)
C2—C3	1.381 (4)	C15—H15A	0.9700
C2—H2	0.9300	C15—H15B	0.9700
C3—C4	1.360 (4)	C16—H16A	0.9700
C3—H3	0.9300	C16—H16B	0.9700
C4—C5	1.342 (5)	C17—C18	1.478 (3)
C4—H4	0.9300	C18—C19	1.376 (3)
C5—C6	1.382 (4)	C18—C23	1.377 (3)
C5—H5	0.9300	C19—C20	1.379 (4)
C6—H6	0.9300	C19—H19	0.9300
C7—C12	1.369 (4)	C20—C21	1.364 (4)
C7—C8	1.389 (4)	C20—H20	0.9300
C8—C9	1.384 (5)	C21—C22	1.379 (4)
C8—H8	0.9300	C21—H21	0.9300
C9—C10	1.351 (5)	C22—C23	1.375 (3)
C9—H9	0.9300	C22—H22	0.9300
C10—C11	1.340 (5)	C23—C24	1.479 (3)
			
C13—Si1—C7	109.25 (14)	H13A—C13—H13B	109.5
C13—Si1—C14	110.53 (12)	Si1—C13—H13C	109.5
C7—Si1—C14	109.68 (11)	H13A—C13—H13C	109.5
C13—Si1—C1	109.56 (13)	H13B—C13—H13C	109.5
C7—Si1—C1	108.56 (10)	C15—C14—Si1	114.37 (15)
C14—Si1—C1	109.22 (11)	C15—C14—H14A	108.7
C24—N1—C17	111.09 (19)	Si1—C14—H14A	108.7
C24—N1—C16	124.89 (18)	C15—C14—H14B	108.7
C17—N1—C16	123.95 (18)	Si1—C14—H14B	108.7
C2—C1—C6	115.9 (2)	H14A—C14—H14B	107.6
C2—C1—Si1	122.39 (19)	C16—C15—C14	112.66 (18)
C6—C1—Si1	121.7 (2)	C16—C15—H15A	109.1
C1—C2—C3	121.5 (3)	C14—C15—H15A	109.1
C1—C2—H2	119.3	C16—C15—H15B	109.1
C3—C2—H2	119.3	C14—C15—H15B	109.1
C4—C3—C2	120.8 (3)	H15A—C15—H15B	107.8
C4—C3—H3	119.6	N1—C16—C15	112.92 (18)
C2—C3—H3	119.6	N1—C16—H16A	109.0
C5—C4—C3	119.8 (3)	C15—C16—H16A	109.0
C5—C4—H4	120.1	N1—C16—H16B	109.0
C3—C4—H4	120.1	C15—C16—H16B	109.0
C4—C5—C6	120.1 (3)	H16A—C16—H16B	107.8
C4—C5—H5	120.0	O1—C17—N1	123.9 (2)
C6—C5—H5	120.0	O1—C17—C18	129.1 (2)
C5—C6—C1	122.0 (3)	N1—C17—C18	106.93 (18)
C5—C6—H6	119.0	C19—C18—C23	121.4 (2)
C1—C6—H6	119.0	C19—C18—C17	131.2 (2)
C12—C7—C8	114.6 (3)	C23—C18—C17	107.33 (19)
C12—C7—Si1	121.12 (19)	C18—C19—C20	117.6 (2)
C8—C7—Si1	124.2 (2)	C18—C19—H19	121.2
C9—C8—C7	121.7 (4)	C20—C19—H19	121.2
C9—C8—H8	119.2	C21—C20—C19	121.1 (3)
C7—C8—H8	119.2	C21—C20—H20	119.5
C10—C9—C8	121.0 (3)	C19—C20—H20	119.5
C10—C9—H9	119.5	C20—C21—C22	121.4 (3)
C8—C9—H9	119.5	C20—C21—H21	119.3
C11—C10—C9	118.9 (4)	C22—C21—H21	119.3
C11—C10—H10	116.0	C23—C22—C21	117.8 (3)
C9—C10—H10	125.0	C23—C22—H22	121.1
C10—C11—C12	120.2 (4)	C21—C22—H22	121.1
C10—C11—H11	119.9	C22—C23—C18	120.7 (2)
C12—C11—H11	119.9	C22—C23—C24	131.1 (2)
C7—C12—C11	123.6 (3)	C18—C23—C24	108.15 (19)
C7—C12—H12	118.2	O2—C24—N1	124.2 (2)
C11—C12—H12	118.2	O2—C24—C23	129.3 (2)
Si1—C13—H13A	109.5	N1—C24—C23	106.49 (19)
Si1—C13—H13B	109.5		
			
C13—Si1—C1—C2	145.6 (2)	Si1—C14—C15—C16	172.75 (17)
C7—Si1—C1—C2	−95.1 (2)	C24—N1—C16—C15	−98.5 (3)
C14—Si1—C1—C2	24.4 (2)	C17—N1—C16—C15	78.4 (3)
C13—Si1—C1—C6	−35.4 (3)	C14—C15—C16—N1	178.08 (18)
C7—Si1—C1—C6	83.8 (2)	C24—N1—C17—O1	179.8 (2)
C14—Si1—C1—C6	−156.6 (2)	C16—N1—C17—O1	2.5 (3)
C6—C1—C2—C3	−0.6 (4)	C24—N1—C17—C18	−0.7 (2)
Si1—C1—C2—C3	178.4 (2)	C16—N1—C17—C18	−177.91 (18)
C1—C2—C3—C4	0.6 (5)	O1—C17—C18—C19	−1.8 (4)
C2—C3—C4—C5	−0.1 (5)	N1—C17—C18—C19	178.6 (2)
C3—C4—C5—C6	−0.4 (5)	O1—C17—C18—C23	−179.6 (2)
C4—C5—C6—C1	0.3 (4)	N1—C17—C18—C23	0.9 (2)
C2—C1—C6—C5	0.2 (4)	C23—C18—C19—C20	−0.2 (4)
Si1—C1—C6—C5	−178.8 (2)	C17—C18—C19—C20	−177.7 (2)
C13—Si1—C7—C12	173.6 (2)	C18—C19—C20—C21	−0.1 (4)
C14—Si1—C7—C12	−65.2 (2)	C19—C20—C21—C22	0.4 (5)
C1—Si1—C7—C12	54.1 (2)	C20—C21—C22—C23	−0.5 (5)
C13—Si1—C7—C8	−3.2 (3)	C21—C22—C23—C18	0.3 (4)
C14—Si1—C7—C8	118.1 (3)	C21—C22—C23—C24	178.8 (3)
C1—Si1—C7—C8	−122.6 (3)	C19—C18—C23—C22	0.1 (4)
C12—C7—C8—C9	0.1 (5)	C17—C18—C23—C22	178.1 (2)
Si1—C7—C8—C9	177.1 (3)	C19—C18—C23—C24	−178.8 (2)
C7—C8—C9—C10	1.2 (7)	C17—C18—C23—C24	−0.8 (2)
C8—C9—C10—C11	−1.6 (7)	C17—N1—C24—O2	−179.6 (3)
C9—C10—C11—C12	0.6 (6)	C16—N1—C24—O2	−2.4 (4)
C8—C7—C12—C11	−1.2 (4)	C17—N1—C24—C23	0.2 (3)
Si1—C7—C12—C11	−178.2 (2)	C16—N1—C24—C23	177.42 (18)
C10—C11—C12—C7	0.9 (5)	C22—C23—C24—O2	1.4 (5)
C13—Si1—C14—C15	52.0 (2)	C18—C23—C24—O2	−179.9 (3)
C7—Si1—C14—C15	−68.50 (19)	C22—C23—C24—N1	−178.4 (3)
C1—Si1—C14—C15	172.63 (15)	C18—C23—C24—N1	0.4 (3)

### Hydrogen-bond geometry (Å, °)

**Table:**

*D*—H···*A*	*D*—H	H···*A*	*D*···*A*	*D*—H···*A*
C4—H4···O2^i^	0.93	2.63	3.366 (4)	137
C14—H14A···O1^ii^	0.97	2.63	3.582 (3)	169
C19—H19···O2^iii^	0.93	2.51	3.310 (3)	144
C22—H22···O1^iv^	0.93	2.32	3.200 (3)	157

Symmetry codes: (i) −*x*+1, −*y*+1, −*z*; (ii) −*x*+1/2, −*y*+3/2, −*z*; (iii) −*x*+1/2, *y*+1/2, −*z*+1/2; (iv) −*x*+1/2, *y*−1/2, −*z*+1/2.
